# Pneumococcal and influenza vaccination coverage in patients with inflammatory rheumatic diseases receiving immunosuppressive therapy and annual nurse-led education

**DOI:** 10.1093/rap/rkag075

**Published:** 2026-07-06

**Authors:** Françoise Fayet, Céline Lambert, Angélique Fan, Marie-Hélène Salembien, Coralie Yvars, Malory Rodere, Carine Savel, Elodie Vandemeulebroucke, Marion Guittard, Natacha Mrozek, Anne Tournadre, Martin Soubrier

**Affiliations:** Rheumatology Department, CHU Clermont-Ferrand, Clermont-Ferrand, France; Biostatistics Unit, DRCI, CHU Clermont-Ferrand, Clermont-Ferrand, France; Rheumatology Department, CHU Clermont-Ferrand, Clermont-Ferrand, France; Rheumatology Department, CHU Clermont-Ferrand, Clermont-Ferrand, France; Rheumatology Department, CHU Clermont-Ferrand, Clermont-Ferrand, France; Rheumatology Department, CHU Clermont-Ferrand, Clermont-Ferrand, France; Rheumatology Department, CHU Clermont-Ferrand, Clermont-Ferrand, France; Rheumatology Department, CHU Clermont-Ferrand, Clermont-Ferrand, France; Rheumatology Department, CHU Clermont-Ferrand, Clermont-Ferrand, France; Infectious and Tropical Diseases Department, CHU Clermont-Ferrand, Clermont-Ferrand, France; Rheumatology Department, CHU Clermont-Ferrand, Clermont-Ferrand, France; Rheumatology Department, CHU Clermont-Ferrand, Clermont-Ferrand, France

**Keywords:** therapeutic patient education nurse, rheumatoid arthritis, spondyloarthritis, pneumococcal vaccine, influenza vaccine, immunosuppressive therapies

## Abstract

**Objectives:**

Vaccination rates among patients with RA or SpA remain suboptimal. This study examined the uptake of pneumococcal and influenza vaccines among patients undergoing immunosuppressive therapy and receiving annual nurse-led education.

**Methods:**

Patients with RA or SpA who initiated their first biologic therapy between 2015 and 2017 and attended yearly visits with a rheumatologist and a therapeutic patient education (TPE) nurse over 5 years were included. Adherence to pneumococcal and influenza vaccine recommendations and factors associated with vaccine uptake were assessed.

**Results:**

A total of 156 patients were analysed: 60 RA and 96 SpA. Overall, 81% (95% CI 74, 87) adhered to the 5-year pneumococcal vaccination recommendations (82% in RA and 80% in SpA). Among patients with RA, a history of major adverse cardiac events or deep vein thrombosis/pulmonary embolism at baseline and the occurrence of myocardial infarction or stent placement during follow-up were associated with non-adherence. Among patients with SpA, those who experienced infections during follow-up were more likely to adhere to the pneumococcal vaccination recommendations. Between 2015 and 2022, influenza vaccination rates increased from 63% to 73% in RA and from 33% to 47% in SpA. However, only 22% of patients were vaccinated annually over 5 years (22% RA, 22% SpA), while 19% never received this vaccine. Older age was associated with higher influenza vaccination uptake in both groups. In SpA, experiencing infections during follow-up was associated with higher influenza vaccination rates.

**Conclusion:**

Pneumococcal vaccination coverage was high among patients monitored annually by a TPE nurse. Influenza vaccination coverage improved over time but remained insufficient, particularly among patients with SpA.

Key messages

- Information, education and awareness are key determinants of vaccination adherence.
- All healthcare professionals have an important role to play.
- Influenza vaccination rates have improved but remain insufficient, especially among SpA patients.
- Older patients are more likely to receive annual influenza vaccines.

## Introduction

Patients with chronic inflammatory rheumatic (CIR) diseases such as RA and SpA are at an increased risk of infection, particularly due to the use of immunosuppressive therapies, including conventional DMARDs (cDMARDs) and biologic DMARDs (bDMARDs) [1]. Vaccination is a key strategy for preventing infections and their complications in this population [1]. To reduce infection risk, international guidelines recommend vaccination for CIR patients receiving immunosuppressive therapy [1]. In particular, pneumococcal and annual influenza vaccination are recommended for all patients receiving immunosuppressive therapy [1]. Influenza vaccination is administered annually, whereas pneumococcal vaccination requires an initial dose followed by a booster dose after 2 months and further doses every 5 years.

Despite these recommendations, vaccination coverage remains insufficient. The international Comorbidities in Rheumatoid Arthritis (COMORA) and Comorbidities in Spondyloarthritis (COMOSPA) studies, designed to assess comorbidities and adherence to recommendations, reported suboptimal vaccination coverage in these patients [2, 3]. Among the 411 French RA patients included in the COMORA study, 57% had received a pneumococcal vaccination within the previous 5 years and 29% had received an influenza vaccination in the previous year [2]. Among the 337 French SpA patients included in the COMOSPA study, 45% had received a pneumococcal vaccination within the previous 5 years and 37% had received an annual influenza vaccination [3]. In another French cohort of 584 CIR patients receiving biologic treatment, vaccination coverage was 62% for pneumococcus and 44% for influenza [4]. Similar results have been reported in other European surveys [5–10]. These concerning findings highlight the need for strategies to improve vaccination coverage. Therapeutic patient education (TPE), combined with regular follow-up and holistic care, may enhance adherence [4–6]. Rheumatology nurses and TPE have an important role in patient monitoring. European recommendations emphasise their role in the overall management of chronic inflammatory arthritis [11] and in delivering TPE to patients with inflammatory arthritis [12].

First, we evaluated pneumococcal and influenza vaccination coverage in a cohort of CIR patients receiving immunosuppressive therapy and annual nurse-led education. Second, we investigated factors associated with vaccination adherence among RA and SpA patients.

## Methods

### Study design and population

Patients >18 years of age with active RA or SpA who attended a French rheumatology department of the University Hospital were invited to participate in the longitudinal Cardiovascular Risk and Chronic Inflammatory Rheumatism (RCVRIC) cohort (RCVRIC AOI 2014 N° ID-RCB-A01847-40). Patients met the 2010 RA classification criteria [13] and the Assessment of SpondyloArthritis international Society criteria for SpA [14, 15]. Among these, we included those initiating biologic therapy. The choice of treatment was at the rheumatologist’s discretion. All patients underwent annual follow-up visits with both their rheumatologist and a TPE nurse. Patients enrolled between 2015 and 2017 who had at least 5 years of follow-up and at least one TPE nurse consultation per year were included in the analysis (Supplementary Fig. S1). The study was approved by the local ethics committee of Clermont-Ferrand University Hospital (CPP Sud-Est 6, ref: AU1161) and all patients provided informed consent.

### Data collection

Demographic and clinical data, including medical history, were collected at baseline. Treatments received within 6 months of inclusion were recorded. Pulmonary, cardiovascular, cancer-related and infectious events were recorded during the 5-year follow-up period. Adherence to pneumococcal vaccination recommendations was defined as receiving one dose of the 13-valent pneumococcal conjugate vaccine (PCV13), followed by one dose of the 23-valent pneumococcal polysaccharide vaccine (PPSV23) 2 months later, and a further dose 5 years after the initial PPSV23 dose. Pneumococcal vaccinations administered prior to cohort entry were included when assessing adherence. Adherence to influenza vaccination recommendations was defined as receiving a yearly influenza vaccine during the 5-year follow-up period. At baseline, vaccination history was verified using medical records and patient vaccination booklets.

### Nurse-led TPE

The TPE nurse identified and managed comorbidities, including infections, cardiovascular disease, cancer and osteoporosis. Each consultation focused on the presence of comorbidities, associated risk factors (e.g. diabetes, pulmonary disease, smoking, age, weight), adherence to clinical recommendations and patient management and education (e.g. dietary advice, physical activity, lipid-lowering therapy, vaccination status, smoking cessation). To prevent infectious diseases, the vaccination schedule was checked for influenza and pneumococcus. If the vaccination schedule was not up to date, vaccination was offered or advised, with shared decision-making respected. The TPE nurse provided information on the benefits of vaccination and the risks associated with non-vaccination while addressing the patient’s concerns, beliefs and motivations. A report of each consultation was sent to the patient, their general practitioner (GP) and their rheumatologist.

### Statistical analysis

Statistical analyses were performed using Stata version 16 (StataCorp, College Station, TX, USA). All tests were two-sided, with significance set at 0.05. Analyses were conducted separately for patients with RA and SpA. No adjustment for multiple comparisons was applied to secondary analyses, which were considered exploratory [16]. Categorical variables are presented as number and percentage and quantitative variables as mean (s.d.) or median [interquartile range (IQR)], according to their distribution. Adherence to pneumococcal and influenza vaccination recommendations was expressed as rates and 95% CIs, calculated using the binomial distribution. Factors associated with vaccination adherence were studied using the chi-squared test or Fisher’s exact test for categorical variables and Student’s *t*-test or Mann–Whitney test for quantitative variables, as appropriate. For categorical variables, Cramér’s V was calculated to estimate the effect size of the significant association and interpreted as follows: 0.1, small effect; 0.3, medium effect; and 0.5, large effect. The evolution of influenza vaccination rates over time was compared between RA and SpA using a generalised linear mixed model with a logit link function, including pathology (RA or SpA), year (2015–2022) and their interaction (pathology × year) as fixed effects and patient as a random effect.

## Results

A total of 156 patients were included: 60 (38%) with RA and 96 (62%) with SpA. The mean age was 58.0 years (s.d. 11.3) for RA patients and 44.7 years (s.d. 11.2) for SpA patients. Women accounted for 78% of the RA group and 54% of the SpA group. The median disease duration was 4 years (IQR 1–12) for RA and 2 years (IQR 0–14) for SpA. The main characteristics of RA and SpA patients are summarised in Tables 1 and 2, respectively.

**Table 1:** Factors associated with adherence to the pneumococcal vaccination schedule (5-year revaccination) and the annual influenza vaccination recommendations in RA patients.

Characteristics	Total (*N* = 60)	Pneumococcal vaccination			Influenza vaccination		
		Non-adherent (*n* = 11)	Adherent (*n* = 49)	*P*-value	Non-adherent (*n* = 47)	Adherent (*n* = 13)	*P*-value
**Baseline sociodemographic**							
** Age, years, mean (s.d.)**	58.0 (11.3)	57.5 (12.4)	58.2 (11.2)	0.87	56.2 (11.7)	64.8 (6.8)	**0.002**
** Female, *n* (%)**	47 (78)	9 (82)	38 (78)	1.00	36 (77)	11 (85)	0.71
** Body mass index, kg/m^2^, mean (s.d.)**	26.4 (5.3)	27.4 (5.2)	26.2 (5.3)	0.47	26.2 (5.6)	27.0 (3.7)	0.57
** Post-secondary education, *n*/*N* (%)**	11/33 (33)	2/5 (40)	9/28 (32)	1.00	6/24 (25)	5/9 (56)	0.12
** Disease duration, years, median (IQR)**	4 (1–12)	4 (1–13)	4 (2–11)	0.92	4 (2–11)	4 (1–15)	0.93
** Smoking (actual or past), *n* (%)**	27 (45)	3 (27)	24 (49)	0.32	20 (43)	7 (54)	0.47
** Daily alcohol consumption, *n* (%)**	13 (22)	1 (9)	12 (24)	0.43	10 (21)	3 (23)	1.00
**Baseline clinical**							
** DAS28-ESR (*n* = 59), mean (s.d.)**	4.1 (1.1)	4.3 (0.8)	4.1 (1.2)	0.46	4.0 (1.2)	4.5 (1.0)	0.23
** DAS28-CRP (*n* = 59), mean (s.d.)**	4.0 (1.0)	4.2 (0.8)	4.0 (1.1)	0.48	4.0 (1.0)	4.1 (1.0)	0.71
** CDAI (*n* = 59), mean (s.d.)**	18.3 (8.1)	17.8 (6.9)	18.4 (8.4)	0.81	18.0 (8.3)	19.3 (7.6)	0.58
** SDAI (*n* = 59), mean (s.d.)**	20.2 (10.8)	20.5 (8.5)	20.1 (11.4)	0.91	20.1 (11.6)	20.5 (7.7)	0.91
** HAQ (*n* = 48), median (IQR)**	0.81 (0.57–1.25)	0.88 (0.75–1.63)	0.81 (0.50–1.25)	0.40	1.00 (0.57–1.25)	0.75 (0.57–1.75)	0.65
** RAID (*n* = 45), mean (s.d.)**	5.4 (2.0)	5.4 (1.6)	5.4 (2.1)	0.72	5.4 (2.0)	5.5 (2.2)	0.73
**Treatments taken during the 6 months following inclusion, *n* (%)**							
** bDMARD**				1.00			0.18
** TNF inhibitor**	42 (70)	8 (73)	34 (69)		35 (74)	7 (54)	
** Other**	18 (30)	3 (27)	15 (31)		12 (26)	6 (46)	
** csDMARD**	56 (93)	10 (91)	46 (94)	0.57	44 (94)	12 (92)	1.00
** Methotrexate**	49 (82)	9 (82)	40 (82)	1.00	38 (81)	11 (85)	1.00
** Corticosteroids**	30 (50)	5 (45)	25 (51)	0.74	26 (55)	4 (31)	0.12
**Comorbidities at baseline, *n* (%)**							
** Cardiovascular disease**	25 (42)	6 (55)	19 (39)	0.50	19 (40)	6 (46)	0.71
** MACE**	5 (8)	3 (27)	2 (4)	0.04	4 (9)	1 (8)	1.00
** MI or stents**	2 (3)	1 (9)	1 (2)	0.34	2 (4)	0 0	1.00
** Stroke**	0 0	0 0	0 0	NA	0 0	0 0	NA
** Heart failure**	3 (5)	2 (18)	1 (2)	0.08	2 (4)	1 (8)	0.53
** Hypertension**	24 (40)	5 (45)	19 (39)	0.74	19 (40)	5 (38)	0.90
** Diabetes**	2 (3)	0 0	2 (4)	1.00	1 (2)	1 (8)	0.39
** Kidney failure**	2 (3)	0 0	2 (4)	1.00	2 (4)	0 0	1.00
** DVT and pulmonary embolism**	2 (3)	2 (18)	0 0	0.03	1 (2)	1 (8)	0.39
** Pulmonary disease**	6 (10)	2 (18)	4 (8)	0.30	4 (9)	2 (15)	0.60
** Respiratory failure**	1 (2)	1 (9)	0 0	0.18	0 0	1 (8)	0.22
** COPD or chronic bronchitis**	1 (2)	1 (9)	0 0	0.18	0 0	1 (8)	0.22
** Asthma**	5 (8)	1 (9)	4 (8)	1.00	4 (9)	1 (8)	1.00
** Pulmonary fibrosis**	1 (2)	1 (9)	0 0	0.18	0 0	1 (8)	0.22
** Cancer**	5 (8)	1 (9)	4 (8)	1.00	4 (9)	1 (8)	1.00
** Hospitalisation for infection**	3 (5)	0 0	3 (6)	1.00	3 (6)	0 (0.0)	1.00
**Events occurring during follow-up, *n* (%)**							
** Cardiovascular events**	15 (25)	4 (36)	11 (22)	0.44	12 (26)	3 (23)	1.00
** MACE**	4 (7)	2 (18)	2 (4)	0.15	3 (6)	1 (8)	1.00
** MI or stents**	2 (3)	2 (18)	0 0	0.03	1 (2)	1 (8)	0.39
** Stroke**	1 (2)	0 0	1 (2)	1.00	1 (2)	0 0	1.00
** Heart failure**	1 (2)	0 0	1 (2)	1.00	1 (2)	0 0	1.00
** Hypertension**	7 (12)	1 (9)	6 (12)	1.00	6 (13)	1 (8)	1.00
** Diabetes**	2 (3)	1 (9)	1 (2)	0.34	1 (2)	1 (8)	0.39
** Kidney failure**	7 (12)	2 (18)	5 (10)	0.60	4 (9)	3 (23)	0.17
** DVT and pulmonary embolism**	0 0	0 0	0 0	NA	0 0	0 0	NA
** Cancer**	3 (5)	0 0	3 (6)	1.00	2 (4)	1 (7)	0.53
** Infectious events**	24 (40)	5 (45)	19 (39)	0.74	19 (40)	5 (38)	0.90
** Respiratory infectious events**	18 (30)	3 (27)	15 (31)	1.00	14 (30)	4 (31)	1.00
** Hospitalisation for infection**	5 (8)	1 (9)	4 (8)	1.00	5 (11)	0 0	0.58

DAS28: 28-joint disease activity score; CDAI: Clinical Disease Activity Index; SDAI: Simplified Disease Activity Index; RAID: rheumatoid arthritis impact of disease; csDMARD: conventional synthetic DMARD; DVT: deep vein thrombosis; COPD: chronic obstructive pulmonary disease.

In the event of missing data, the numbers are specified in parentheses in the first column. *P*-values in bold are <0.05.

**Table 2:** Factors associated with adherence to the pneumococcal vaccination schedule (5-year revaccination) and the annual influenza vaccination recommendations in SpA patients.

Characteristics	Total (*n* = 96)	Pneumococcal vaccination			Influenza vaccination		
		Non-adherent (*n* = 19)	Adherent (*n* = 77)	*P*-value	Non-adherent (*n* = 75)	Adherent (*n* = 21)	*P*-value
**Baseline sociodemographic**							
** Age, years, mean (s.d.)**	44.7 (11.2)	42.1 (11.8)	45.3 (11.1)	0.30	43.0 (10.8)	50.8 (10.9)	**0.004**
** Female, *n* (%)**	52 (54)	12 (63)	40 (52)	0.38	42 (56)	10 (48)	0.50
** Body mass index, kg/m^2^, mean (s.d.)**	27.1 (5.9)	26.9 (6.2)	27.2 (5.9)	0.86	26.9 (5.6)	27.9 (6.9)	0.53
** Post-secondary education, *n*/*N* (%)**	30/64 (47)	7/15 (47)	23/49 (47)	0.99	23/51 (45)	7/13 (54)	0.57
** Disease duration, years, median (IQR)**	2 (0–14)	2 (1–5)	3 (0–16)	0.37	2 (0–14)	5 (0–14)	0.61
** Smoking (actual or past), *n* (%)**	60 (62)	11 (58)	49 (64)	0.64	48 (64)	12 (57)	0.57
** Daily alcohol consumption, *n*/*N* (%)**	13/95 (14)	2 (11)	11/76 (14)	1.00	9 (12)	4/20 (20)	0.46
**Baseline clinical characteristics**							
** BASDAI (*n* = 79), *n* (%)**	5.7 (1.4)	5.8 (1.7)	5.7 (1.3)	0.77	5.7 (1.3)	5.7 (1.6)	0.87
** ASDAS-CRP (*n* = 95), *n* (%)**	3.2 (0.8)	3.2 (0.8)	3.2 (0.8)	0.98	3.3 (0.8)	3.0 (0.8)	0.14
** HAQ-AS (*n* = 77), median (IQR)**	0.75 (0.63–1.13)	0.88 (0.75–1.00)	0.75 (0.63–1.13)	0.55	0.75 (0.63–1.00)	0.75 (0.63–1.25)	0.70
**Treatments taken during the 6 months following inclusion, *n* (%)**							
** bDMARD**				0.60			0.33
** TNF inhibitor**	90 (94)	19 (100)	71 (92)		69 (92)	21 (100)	
** Other**	6 (6)	0 0	6 (8)		6 (8)	0 0	
** csDMARD**	27 (28)	6 (32)	21 (27)	0.71	21 (28)	6 (29)	0.96
** Methotrexate**	21 (22)	6 (32)	15 (19)	0.35	19 (25)	2 (10)	0.15
** Corticosteroids**	3 (3)	0 0	3 (4)	1.00	2 (3)	1 (5)	0.53
**Comorbidities at baseline, *n* (%)**							
** Cardiovascular disease**	23 (24)	4 (21)	19 (25)	1.00	14 (19)	9 (43)	**0.02**
** MACE**	2 (2)	0 0	2 (3)	1.00	1 (1)	1 (5)	0.39
** MI or stents**	2 (2)	0 0	2 (3)	1.00	1 (1)	1 (5)	0.39
** Stroke**	0 0	0 0	0 0	NA	0 0	0 0	NA
** Heart failure**	0 0	0 0	0 0	NA	0 0	0 0	NA
** Hypertension**	19 (20)	3 (16)	16 (21)	0.76	11 (15)	8 (38)	**0.03**
** Diabetes**	5 (5)	0 0	5 (6)	0.58	2 (3)	3 (14)	0.07
** Kidney failure**	1 (1)	1 (5)	0 0	0.20	1 (1)	0 0	1.00
** DVT and pulmonary embolism**	2 (2)	0 0	2 (3)	1.00	2 (3)	0 0	1.00
** Pulmonary disease**	8 (8)	2 (11)	6 (8)	0.66	8 (11)	0 0	0.19
** Respiratory failure**	0 0	0 0	0 0	NA	0 0	0 0	NA
** COPD or chronic bronchitis**	1 (1)	0 0	1 (1)	1.00	1 (1)	0 0	1.00
** Asthma**	6 (6)	2 (11)	4 (5)	0.34	6 (8)	0 0	0.33
** Pulmonary fibrosis**	1 (1)	0 0	1 (1)	1.00	1 (1)	0 0	1.00
** Cancer**	2 (2)	0 0	2 (3)	1.00	2 (3)	0 0	1.00
** Hospitalisation for infection**	4 (4)	0 0	4 (5)	0.58	3 (4)	1 (5)	1.00
**Events occurring during follow-up, *n* (%)**							
** Cardiovascular events**	12 (12)	2 (11)	10 (13)	1.00	10 (13)	2 (10)	1.00
** MACE**	4 (4)	0 0	4 (5)	0.58	3 (4)	1 (5)	1.00
** MI or stents**	3 (3)	0 0	3 (4)	1.00	2 (3)	1 (5)	0.53
** Stroke**	1 (1)	0 0	1 (1)	1.00	1 (1)	0 0	1.00
** Heart failure**	1 (1)	0 0	1 (1)	1.00	1 (1)	0 0	1.00
** Hypertension**	4 (4)	0 0	4 (5)	0.58	3 (4)	1 (5)	1.00
** Diabetes**	3 (3)	1 (5)	2 (3)	0.49	2 (3)	1 (5)	0.53
** Kidney failure**	4 (4)	1 (5)	3 (4)	1.00	4 (5)	0 0	0.57
** DVT and pulmonary embolism**	0 0	0 0	0 0	NA	0 0	0 0	NA
** Cancer**	6 (6)	1 (5)	5 (6)	1.00	5 (7)	1 (5)	1.00
** Infectious events**	36 (37)	3 (16)	33 (43)	**0.03**	23 (31)	13 (62)	**0.009**
** Respiratory infectious events**	23 (24)	2 (11)	21 (27)	0.15	13 (17)	10 (48)	**0.004**
** Hospitalisation for infection**	2 (2)	1 (5)	1 (1)	0.36	2 (3)	0 0	1.00

ASDAS: Ankylosing Spondylitis Disease Activity Score; HAQ-AS: Health Assessment Questionnaire for Ankylosing Spondylitis; csDMARD: conventional synthetic DMARD; COPD: chronic obstructive pulmonary disease.

In the event of missing data, the numbers are specified in parentheses in the first column.

*P*-values in bold are <0.05.

Adherence to the 5-year pneumococcal vaccination recommendations was 81% overall (95% CI 74, 87): 82% in patients with RA and 80% in patients with SpA. Among patients with RA, a history of major adverse cardiovascular events (MACE) (Cramér’s V = −0.32) or deep vein thrombosis/pulmonary embolism at baseline (Cramér’s V = −0.39), as well as the occurrence of myocardial infarction (MI) during follow-up (Cramér’s V = −0.39), were more frequently observed among those less likely to adhere to pneumococcal vaccination recommendations (Table 1). Among patients with SpA, the occurrence of an infectious event within the 5-year follow-up was more frequent (Cramér’s V = 0.22) in patients who adhered to the pneumococcal vaccination recommendations (43%) than in those who did not (16%) (Table 2).

**Figure 1:**
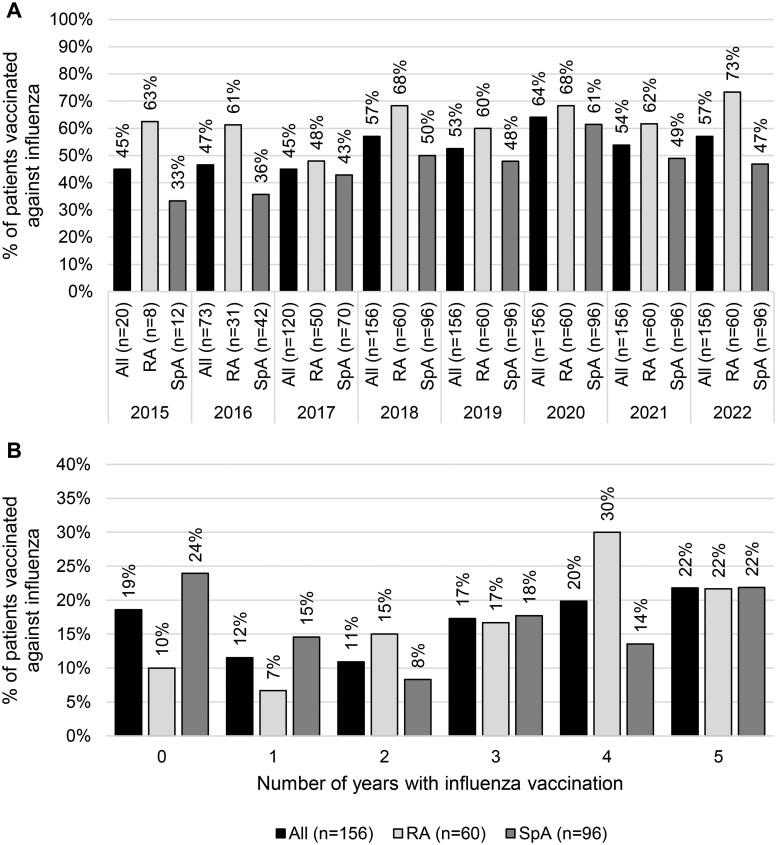
The yearly influenza vaccination rates for the total population and for specific pathologies **(A)** between 2015 and 2022 and **(B)** adherence to annual vaccinations over a 5-year follow-up period

Over time, influenza vaccination rates increased in both groups, with no significant difference in trends between RA and SpA (pathology × year interaction, *P* = 0.62). In the RA group, the proportion of vaccinated patients increased from 63% (*n* = 5/8) in 2015 to 73% (*n* = 44/60) in 2022. In the SpA group, it increased from 33% (*n* = 4/12) to 47% (*n* = 45/96) over the same period >(Fig. 1A).

During the 5-year follow-up period, only 22% of patients (95% CI 16, 29) received an annual influenza vaccination (22% RA, 22% SpA), while 19% were never vaccinated (Fig. 1B). The proportion of patients who had never received the influenza vaccine was significantly higher in the SpA group than in the RA group (24% *vs* 10%, *P* = 0.03).

Older age was significantly associated with better adherence to annual influenza vaccination recommendations in both groups. RA patients vaccinated annually were older than those who were not [64.8 (s.d. 6.8) *vs* 56.2 (s.d. 11.7) years, *P* = 0.002; Table 1]. Similarly, SpA patients vaccinated annually were older than those who were not [50.8 (s.d. 10.9) *vs* 43.0 (s.d. 10.8) years, *P* = 0.004; Table 2]. Among patients with SpA, annual influenza vaccination was also significantly associated with experiencing an infection during the 5-year follow-up period: 62% of patients vaccinated annually had experienced such events compared with 31% of those not vaccinated annually (*P* = 0.009), particularly respiratory infections (48% *vs* 17%, *P* = 0.004; Table 2).

## Discussion

After 5 years of structured follow-up, the pneumococcal vaccination coverage rate was remarkably high among a cohort of CIR patients receiving immunosuppressive therapy and annual nurse-led education as compared with previously published data [2–10]. In contrast, influenza vaccination coverage remained suboptimal and far below the World Health Organization’s target of 75%. The lower influenza vaccination rate observed in patients with SpA is likely related to their younger age and fewer comorbidities, which may reduce their perceived susceptibility to severe infections. Similar to other studies [4–5, 10], we found higher influenza vaccination rates in older patients. Nonetheless, there was no significant difference in the evolution of influenza vaccination coverage between RA and SpA, suggesting that repeated nurse-led follow-up and education benefit both groups.

The central role of nurses in optimising vaccination practices is supported by previous randomised controlled studies. In the Comorbidities and Education in Rheumatoid Arthritis (COMEDRA) study, involving 970 RA patients, influenza vaccination rates were significantly higher in patients followed by a nurse compared with those followed exclusively by a rheumatologist (39% *vs* 22%, *P* = 0.005), while pneumococcal vaccination rates were 11% *vs* 6%, respectively [17]. Similarly, the Comorbidities and Education in Spondyloarthritis (COMEDSPA) study of 502 patients with SpA reported higher influenza and pneumococcal vaccination rates in the nurse-led follow-up arm (29% and 40%) than in the rheumatologist-only follow-up arm (10% and 21%, *P* < 0.05) [18]. Brocq *et al.* [4] also reported higher vaccination rates when both rheumatologists and GPs were involved in providing information to patients (88% for pneumococcal, 74% for influenza) than when a single professional provided information or when no information was provided. Costas *et al.* [9] showed that only 40% of patients fully understood the benefits of pneumococcal vaccination, yet this knowledge and risk awareness strongly correlated with vaccine uptake (*P* < 0.001). Likewise, Moraliyska *et al.* [8] showed that discussions with a rheumatologist markedly increased the likelihood of receiving influenza [odds ratio (OR) 12.9] and pneumococcal (OR 32.5) vaccinations. Altogether, these studies underline that all healthcare professionals contribute to improving adherence and that information, education and awareness are key determinants of vaccination behaviour [4, 8–9, 19].

Rheumatology TPE nurses collaborate closely with patients, rheumatologists and GPs, with a shared focus on optimising care and outcomes [11, 12]. According to data from the French public health system, flu vaccination rates in the general population have also increased as a result of the enhanced vaccination strategy implemented in response to the COVID-19 pandemic (from 46% to 53% in the same period). Pneumococcal vaccination is currently one of the least widely adopted among those at risk for pneumococcal infections. Between 2019 and 2022, the pneumococcal vaccination coverage rate in this population also increased, from 8% to 12%. Although our study did not collect reasons for non-vaccination, previous qualitative research has identified fear of side effects and perceived lack of relevance—particularly among younger patients—as major barriers [20]. A further limitation of our study is its limited statistical power, reflecting the small proportion of patients who were non-adherent with pneumococcal vaccination and those fully adherent to annual influenza vaccination. Non-significant results should therefore be interpreted with caution, as insufficient statistical power may have precluded the detection of true associations. On the other hand, the descriptive nature of the study and the absence of a comparator group without nurse-led follow-up limit the ability to formally assess the causal effect of nurse-led education.

## Conclusion

Our study demonstrated high pneumococcal vaccination rates and an increase in influenza vaccination among patients who received nurse-led education. However, influenza vaccination coverage remained low among patients with SpA despite this intervention. Motivational interviewing could be explored as an additional strategy to further improve vaccination uptake in this population.

## Supplementary Material

rkag075_Supplementary_Data

## Data Availability

While the data are not publicly available, they may be obtained upon reasonable request to the corresponding author.
