# Supplementary material for: Pneumococcal and influenza vaccination coverage in patients with inflammatory rheumatic diseases receiving immunosuppressive therapy and annual nurse-led education
Source: Rheumatol Adv Pract. 2026 Jul 6;10(3):rkag075. doi: 10.1093/rap/rkag075 (PMC13394688; doi:10.1093/rap/rkag075)
Supplement: rkag075_Supplementary_Data [file rkag075_supplementary_data.zip › Supplementary_fig S1_Flow_chart.docx]

Supplementary Figure S1. Flow chart of the study
